# Case Report: Insulinoma Presenting as Excessive Daytime Somnolence

**DOI:** 10.3389/fendo.2021.712392

**Published:** 2021-11-26

**Authors:** Zhennan Yu, Yongliang Wang, Yaqi Sun, Yumei Wang, Yayun Tian, Qinying Ma, Ying Fu

**Affiliations:** Department of Psychiatry, The First Hospital of Hebei Medical University, Shijiazhuang, China

**Keywords:** somnolence, sleep disorder, insulinoma, hypoglycemia, case report

## Abstract

Currently, undiagnosed insulinomas remain a difficult clinical dilemma because its symptoms in most cases can easily be misdiagnosed as other diseases. In this article, we present the case of a 14-year-old girl who presented to our hospital with recurrent episodes of excessive daytime sleepiness and abnormal behavior during sleep that had been going on for 3 months. Insulinoma is a rare neuroendocrine tumor that causes excessive release of insulin, resulting in episodes of hypoglycemia. It usually manifests as autonomic sympathetic symptoms. These symptoms resolved rapidly with the administration of glucose. After successful removal of the tumor, daytime sleepiness and abnormal nighttime behavior of the patient did not reappear.

## Introduction

Insulinoma is the most common functional pancreatic neuroendocrine tumor, with a reported incidence of 0.5–5 per million person-years (1). In over 90% of cases, they are solitary and benign (2). Insulinomas have a male-to-female ratio of 2:3 and can occur at any age. In this article, we report a case of severe excessive daytime sleepiness and abnormal nighttime sleep behavior associated with insulinoma-induced hypoglycemia. Various hypoglycemic symptoms result from excessive insulin production due to tumor proliferation of pancreatic β cells (3). The main symptoms include sympathetic excitation due to hypoglycemia, such as pallor, cold extremities, cold sweats, palpitations, and hand tremors; recurrent episodes of hypoglycemia leading to cortical depression and psychiatric abnormalities; and lack of glucose in brain cells leading to impaired consciousness, such as drowsiness, coma, unresponsiveness, mental retardation, and possibly seizures in severe cases (4, 5).

In this report, we report a case of excessive daytime sleepiness and abnormal behavior during sleep due to hypoglycemia that was seen in our sleep center. In clinical practice, the idea of daytime sleepiness, in addition to narcolepsy and depressive episode, has to be actively broadened, such as in this case, which eventually confirmed its association with a disease related to the endocrine system.

## Case Report

A 14-year-old female patient presented with increased daytime sleepiness with abnormal behavior during sleep for 3 months. The parent of the patient reported that the patient experienced intermittent increased daytime sleepiness since November 2019. She would usually go to bed at 11 o’clock in the evening and could only wake up until the next-day afternoon. Her parent could not wake her up during sleep. Within 30 min after waking up, she was in a twilight state and walked unsteadily. Sometimes, she could not remember where she was, and subsequently, she gradually performed normally. On February 9, 2020, the parent first noticed abnormal behavior during sleep in the girl, which was mainly characterized by the inability to wake up during sleep, eyes rolling upwards, and twitching of the limbs, a process that could last up to 1 h and gradually improved on its own. Therefore, polysomnography (PSG) and continuous polysomnographic latency testing (MSLT) were performed at that time. Nocturnal video PSG disclosed a sleep latency of 56.5 min, REM latency of 8.0 min, sleep efficiency of 83.4%, and abnormal representation of the different sleep stages, with increased N_1_% sleep period time (SPT) of 8.2%, increased N_2_% SPT of 63.7%, decreased N_3_% SPT of 3.2%, and decreased REM% SPT of 24.9%; periodic leg movement during sleep (PLMS) index was 1.4/h. Apnea/hypopnea index was 0.9/h with an average oxygen saturation of 98%. During the next-day MSLT, the patient fell asleep in three of the five sessions with a mean sleep latency of 17.9 min and three times of SOREM sleep occurred in five naps. Her Epworth Sleepiness Scale score was 15 points and her Pittsburgh Sleep Quality Index was 7 points.

Since the diagnosis of the patient was not clear, admission was recommended as soon as possible and diagnosis such as atypical depression, sleep rhythm disturbance, seizures, and idiopathic hypersomnia was excluded. On March 6, 2020, her family explained that over the previous 4 months, the patient had more frequent daytime sleepiness episodes than before, and on March 5, she did not eat or drink all day and had been sleeping, and the family could not wake her up. The general physical examination of the patient revealed the following data: height of 179 cm, weight of 70 kg, and BMI of 21.8 kg/m^2^. The rest of the general and systemic examination was within normal limits. However, on the first day after admission, the laboratory test results revealed serum fasting glucose levels (1.9 mmol/L) at 10:39 a.m. While the physician was verifying the test results of the patient, the family complained that the patient was screaming, had involuntary body twitching, and could not wake up. An intravenous infusion of 50% glucose rapidly reversed her hypoglycemia and gradually restored consciousness.

In order to identify the cause of hypoglycemia, follow-up checks were performed. Continuous glucose monitoring (CGM) also showed that blood glucose level fluctuates between 2 and 4 mmol/L during the day and between 4 and 8 mmol/L at night. Islet cell antibody and anti-glutamate decarboxylase antibody were negative. Thus, she was suspected to be affected by a hypoglycemic disorder and was referred to the Department of General Surgery at Peking Union Medical College Hospital. An 8-h fast produced hypoglycemia (glucose levels of 2.4 mmol/L), with insulin levels of 23.2 μIU/ml, C-peptide levels of 2.48 ng/ml, and proinsulin levels of 1,045 pg/ml. The patient was then examined for insulinoma. A pancreatic head mass of 10 mm was evidenced by abdominal enhanced CT, MRI, and transendoscopic ultrasound. These results led to a diagnosis of insulinoma. After surgical enucleation, it was histopathologically confirmed to be an insulinoma. After 2 months of follow-up, the blood glucose level of the patient normalized, and hypersomnia or abnormal behavior during sleep did not occur again. The Epworth Sleepiness Scale score and the Pittsburgh Sleep Index after the operation were 4 and 5 points, respectively. The patient feels that daytime sleepiness improved significantly, and she has not experienced behavioral abnormalities during sleep once again.

## Discussion

In this study, we reported a case of insulinoma presenting as daytime sleepiness and abnormal behavior during sleep. In the past, several case reports of insulinoma presenting as seizures or paroxysmal disorders have been previously described. The extremely low blood glucose level raised the suspicion that hypersomnia and hypoglycemia were induced by insulinoma. However, only three other cases of insulinoma presenting as hypersomnia have been reported till now (5–7). This case highlights the importance of considering hypoglycemia in hypersomnia.

During the diagnosis of the disease of the patient, we first consider whether there is a possibility of narcolepsy based on the irresistible increase in daytime sleep of the patient. However, according to relevant follow-up checks, MSLT suggested that this patient had a shortened REM latency and three times of SOREM sleep. The current study found that narcolepsy is associated with a selective deficit of appetite-producing neurons in the posterior hypothalamus. Hypocretin is a neuropeptide of hypothalamic origin that promotes wakefulness and inhibits REM sleep. Hypocretin also plays critical roles in the regulation of feeding, wakefulness, reward system, energy homeostasis, and other physiological processes (8). Ablation of orexin neurons in mice results in narcolepsy, hypoactivity, and late-onset obesity with or without hypophagia (9). Thus, impairment of the hypocretin system results in inappropriate intrusion of REM sleep, leading to episodes of sleepiness and cataplexy. One study found that a subject with anti-Ma2-associated encephalitis and narcolepsy showed reduced sleep efficiency on nocturnal polysomnography and shortened mean sleep latency and sleep onset REM periods on MSLT, along with monitoring of low cerebrospinal fluid appetite-1 levels (10). This study has hypothesized that when secondary narcolepsy is due to a focal lesion in the hypothalamus, it leads to a decrease in the production of appetitin (10). In our case, the level of appetitin in the cerebrospinal fluid was not tested. However, it is reasonable to infer that the presence of insulinoma leading to prolonged hypoglycemia may have affected the normal function of the hypothalamus to some extent, leading to reduced appetitive hormone secretion and, consequently, to shortened REM latency and increased sleep during SOREM.

Unlike most endocrine pancreatic tumors, the majority (90%) of insulinomas are benign and solitary, and only 10% are malignant. Patients with insulinoma are often free of symptoms despite having critically low glucose levels because of adaptations by the central nervous system to chronic hypoglycemia (11), so the diagnosis is difficult and is commonly delayed mainly because of their unaware or non-specific symptoms. We should note that the unspecific symptoms such as headache, disorientation, seizure, confusion, and irrational behavior in children—especially in the mornings after fasting through the night—might be due to hypoglycemia (12, 13), and it is important to check Whipple’s triad to rule out an insulinoma. It is considered positive if Whipple’s triad is present, and this is defined as a serum glucose level below 2.8 mmol/L, the presence of neuroglycopenic features, and the relief of these symptoms following glucose administration.

Due to the episodic course of the patient, it is easy to be confused with epilepsy. Epilepsy is a chronic disease caused by sudden abnormal discharge of brain neurons, leading to transient brain dysfunction. The clinical manifestations of epileptic seizures are diverse. Most patients can be completely normal during the interictal stage, and epilepsy only appears during the seizure phase. Symptoms related to seizures are mainly convulsions and fainting. Some patients may manifest numbness, dizziness, flushing of the face and body, and so on. EEG is very important for distinguishing epileptic seizures from non-epileptic seizures. Since the PSG examination of the patient did not show epileptic waves and the symptoms improved immediately after glucose infusion, the possibility of epilepsy was ruled out. There are also a few patients who have been diagnosed with epilepsy for years, but are eventually found to have secondary epilepsy due to hypoglycemia. So the seizures eventually disappear when the patient's hypoglycemia symptoms improve.

In insulinomas, hypoglycemic symptoms, hypoglycemia, and hyperinsulinism coexist. If the blood glucose level is <40 mg/dl and the insulin level >6 µIU/ml, it is diagnosed as hypoglycemia due to hyperinsulinism (14). High levels of C-peptide indicate endogenous insulin secretion.

After confirmation of biochemical diagnosis, the exact preoperative anatomic localization of insulinoma needs imaging techniques. Ultrasonography (abdominal/endoscopic), CT scan, or MRI is imperative before a major pancreatic surgery (14). The sensitivities of transabdominal ultrasonography and abdominal CT scanning are low (between 23% and 63% and between 40% and 73%, respectively) as diagnostic procedures. On the other hand, endoscopic ultrasonography has a sensitivity of 95% in the hands of skilled operators. Surgical excision is the treatment of choice for insulinomas. The nature of surgery depends on the size, location, and resectability of the tumors. In small tumors, simple enucleation or distal pancreatectomy could be done. Now, laparoscopic resection is gaining popularity (15).

In summary, our patient experienced episodes of hypersomnia induced by a pancreatic insulinoma. Although pancreatic insulinoma is a benign and curable tumor, it can be fatal if misdiagnosed. Hence, the aim of the present case report is to raise awareness among physicians to consider insulinoma in the differential diagnosis of hypersomnia in patients without other known diseases.

## Data Availability Statement

The original contributions presented in the study are included in the article/supplementary material. Further inquiries can be directed to the corresponding author.

## Ethics Statement

Written informed consent was obtained from the legal guardian/next of kin of the patient for the publication of any potentially identifiable images or data included in this article.

## Author Contributions

ZY wrote the case report. YLW shared his psychiatry expertise in the project. YMW is the head of the research and provided expert supervision. YS and YT contributed to the literature review. QM provided neurology expertise in the project. YF shared his expertise on electromyelography. All authors contributed to the article and approved the submitted version.

## Funding

National Natural Science Foundation of China project number(Number: 81771463).

## Conflict of Interest

The authors declare that the research was conducted in the absence of any commercial or financial relationships that could be construed as a potential conflict of interest.

## Publisher’s Note

All claims expressed in this article are solely those of the authors and do not necessarily represent those of their affiliated organizations, or those of the publisher, the editors and the reviewers. Any product that may be evaluated in this article, or claim that may be made by its manufacturer, is not guaranteed or endorsed by the publisher.
